# Intraoral Drug Delivery: Highly Thiolated κ-Carrageenan as Mucoadhesive Excipient

**DOI:** 10.3390/pharmaceutics15071993

**Published:** 2023-07-20

**Authors:** Gergely Kali, Andrea Fürst, Nuri Ari Efiana, Aida Dizdarević, Andreas Bernkop-Schnürch

**Affiliations:** Center for Chemistry and Biomedicine, Department of Pharmaceutical Technology, Institute of Pharmacy, University of Innsbruck, Innrain 80/82, 6020 Innsbruck, Austria; gergely.kali@uibk.ac.at (G.K.);

**Keywords:** Carrageenan, thiomers, thiolated kappa-carrageenan, mucoadhesion, mucosal residence time

## Abstract

Aim: This study aims to design a novel thiolated κ-carrageenan (κ-CA-SH) and evaluate its potential as an excipient for the design of mucoadhesive drug delivery systems. Methods: Native κ-carrageenan (κ-CA) was thiolated with phosphorous pentasulfide in sulfolane and characterized via ^1^H NMR, FTIR, as well as Ellman’s test. Cytotoxicity was assessed via resazurin assay. In vitro release of the model drug, benzydamine hydrochloride, was determined. Tensile and mucosal residence time studies were performed on buccal and small intestinal mucosa. Mucoadhesive features were investigated via rheological studies with freshly isolated porcine mucus. Results: Thiolated κ-CA (κ-CA-SH) with 1213.88 ± 52 µmol/g thiol groups showed no cytotoxicity at a concentration of 1% (*m*/*v*) and low cytotoxicity up to 2% (*m*/*v*). Benzydamine hydrochloride showed slow release in solution for both polymers. Tensile studies on buccal and intestinal mucosa showed an up to 2.7-fold and 7.7-fold enhancement in the maximum detachment force (MDF) and total work of adhesion (TWA) of κ-CA-SH vs. κ-CA, respectively. The κ-CA-SH exhibited an up to 4.4-fold improved dynamic viscosity with mucus and significantly prolonged residence time on mucosa compared to native κ-CA. Conclusion: Since highly thiolated κ-CA shows a slow release of positively charged active pharmaceutical ingredients and enhanced mucoadhesive properties, it might be a promising excipient for local drug delivery in the oral cavity.

## 1. Introduction

Intraoral drug delivery offers several advantages over the oral route, such as the bypass of first-pass metabolism and rather low enzymatic activity [1]. Hence, buccal administration of active pharmaceutical ingredients (APIs) is an important drug delivery route in order to achieve local or systemic effects [2]. Since a mucus layer covers the oral cavity, mucoadhesive drug delivery systems are beneficial in order to provide intimate contact with the mucosa, prolonging the residence time there [3]. Ionic forces, as well as hydrogen bonding or entanglement of polymer chains with the mucus layer, can trigger mucoadhesion [4]. Moreover, thiolated polymers and oligomers are also highly mucoadhesive as they can form disulfide bonds with cysteine-rich subdomains of mucus glycoproteins [5]. The degree of thiolation greatly affects mucoadhesion. The more thiol groups there are attached to the polymers, the stronger their mucoadhesive properties [6]. For intraoral drug delivery, liquid or semisolid formulations are preferred since solid formulations are rapidly eliminated by the tongue. A sustained drug release from liquid or semisolid formulations containing mucoadhesive polymers, however, is difficult to achieve. The likely most promising strategy to address this challenge is the use of mucoadhesive polymers providing ionic interactions with the drug. In particular, highly charged polymers seem to be advantageous.

Carrageenans (CA) are red algae polysaccharides of high molecular weight formed via alternating units of D-galactose and 3,6-anhydrogalactose by glycosidic bonds [7,8]. Because of sulfate groups in their structure, CAs exhibit a strong anionic character. Depending on the degree as well as the position of these sulfate groups, CAs are classified into either kappa- (κ-), iota- (I-), or lambda- (λ-) CAs. Due to their biocompatibility and biodegradability, CAs have been generally recognized as safe by the Food and Drug Administration since the end of last century and have also been approved by the European Food Safety Authority as food additives [9]. The anionic groups on the backbone of CAs enable the formation of polyelectrolyte complexes with oppositely charged, cationic active pharmaceutical ingredients (APIs), guaranteeing a controlled drug release [9,10]. Besides these advantages, CAs possess mucoadhesive properties that might be even further improved by thiolation [11]. Among others, κ-CA carries a higher amount of hydroxyl groups than, for example, λ-CA, and therefore, a higher degree of thiolation can be reached. Previously, CA was thiolated via a two-step method, resulting in a low thiol content (<180 µmol/g SH) [12].

The aim of this study is to design κ-CA-SH with a high degree of thiolation in order to improve mucoadhesion and to provide sustained release of benzydamine, a cationic model drug. In the following, mucoadhesive features are evaluated by tensile and rheological studies, as well as via the determination of mucosal residence time accompanied by in vitro release studies of benzydamine.

## 2. Material and Methods

### 2.1. Materials

Kappa-carrageenan (κ-CA), phosphorus pentasulfide (P_4_S_10_, 99%), tetramethylene sulfone (sulfolane, 99%), dimethyl sulfoxide-*d_6_* (DMSO-*d_6_*, 99.9%), benzydamine hydrochloride, 5,5′-dithiobis(2-nitrobenzoic acid) (Ellman’s reagent), and sodium borohydride (NaBH_4_, ≥98%), dibutyltin dilaurate (DBTDL) and fluorescein isothiocyanate isomer 1 (FITC, ≥90%) were purchased from Sigma-Aldrich, Wien, Austria. Dialysis tubes of ZelluTrans from Carl Roth, Osterreich, Austria, with MWCO 3500 Da, were used. All the used chemicals were received from commercial sources and used without purification. Porcine small intestine was a donation from a slaughterhouse in Innsbruck.

### 2.2. Synthesis and Purification of Highly Thiolated Kappa-Carrageenan

The κ-CA-SH was synthesized according to our previously published method [13,14]. Briefly, first κ-CA (1.0 g) and phosphorus pentasulfide (4.8 g, 10.6 mmol) were dissolved in 15 mL sulfolane. To this solution, 2 mL of triethylamine (27.0 mmol) was added. The reaction flask was heated up to around 130 °C and stirred overnight. The temperature was dropped to 50 °C, and water was added dropwise to degrade the remaining P_2_S_5_. The product was dialyzed against water at pH 5.5 for 3 days. The title compound was obtained as a light brownish solid.

### 2.3. Characterization of Highly Thiolated Kappa-Carrageenan

^1^H NMR measurements were performed on a “Mars” 400 MHz Avance 4 Neo spectrometer from Bruker Corporation (Billerica, MA, USA, 400 MHz) in dimethyl sulfoxide-*d_6_* (DMSO-*d_6_*). The Fourier-transform infrared (FTIR) spectra of native and per-thiolated CDs were recorded using a Bruker ALPHA FT-IR apparatus equipped with a Platinum ATR (attenuated total reflection) module.

The amount of free thiol groups being attached to the polymer backbone was quantified photometrically using 5,5′-dithiobis(2-nitrobenzoic acid) (Ellman’s reagent), referring to an already established method [15]. The same procedure after reducing disulfide content using sodium borohydride was used to determine the total amount of thiol groups. The amount of thiol groups was calculated employing a calibration curve of increasing concentrations of L-cysteine. Hence, absorbance was measured using a microplate reader at a wavelength of 450 nm (Tecan Spark multimode microplate reader, Grödig, Austria).

Stability of thiol groups at different pH was determined by dissolving 10 mg of polymers in 8 mL of buffer solution (0.1 M acetate and phosphate buffers at pH 5, 6, 7, and 8) at 37 °C under constant shaking at 300 rpm for 4 h.

### 2.4. Evaluation of Cytotoxicity

Evaluating the cytotoxic potential of polymers applying different concentrations ranging from 1% to 4% (*m*/*v*) resazurin assay on Caco-2 cell monolayer was performed [16,17]. In brief, 2.5 × 10^4^ Caco-2 cells per well were seeded to 24-well plates being further cultured for about 14 days at 37 °C in an atmosphere of 5% CO_2_ and 95% relative humidity. Cells treated with 1% (*m*/*v*) Triton X-100 served as positive control regarding cell lysis, while cells with minimum essential medium (MEM) without phenol red are referred to as cell viability being the negative control. After 24 h of incubation, samples were washed with a phosphate-buffered saline solution twice and further incubated with 250 µL of resazurin (44 µM) solution (phosphate-buffered saline and MEM without phenol red 1:20 *v*/*v*) for 2 h. Fluorescence was measured at 540 nm excitation and 590 nm emission wavelengths (Tecan Spark multimode microplate reader, Grödig, Austria). Cell viability was calculated using the following equation:

(1) $$\mathrm{Cell}\;\mathrm{viability}\;[\%]=\frac{average\;fluorescence\;of\;samples}{average\;fluorescence\;of\;cells\;treated\;with\;MEM\;without\;phenol\;red}\;\;\times \;100\;$$

### 2.5. Hemolysis Studies

The damaging effect of κ-CA and κ-CA-SH on the cell membrane of red blood cells was determined in concentrations of 0.1 and 0.2% (*m*/*v*). Briefly, 1 mL of fresh human blood samples from volunteer donors provided by the Landeskrankenhaus-Universitätskliniken (Zentralinstitute für Bluttransfusion und immunologische Abteilung, Innsbruck, Austria) was diluted with 1 mL of sterile HEPES buffer (pH 7.4 with 5% glucose). κ-CA and κ-CA-SH samples in concentrations of 0.1 and 0.2% (*m*/*v*) were incubated with the red blood cells at 37 °C under constant shaking at 300 rpm. Triton™ X-100 (0.5% *v*/*v*) was used as a positive, and sterile HEPES buffer as a negative control. Aliquots (250 μL) were withdrawn after 1 and 3 h, centrifuged at 8000 rpm for 5 min, and the supernatants were analyzed via a microplate reader (Tecan Spark^®^, Tecan Trading AG, Zurich, Switzerland) at 420 nm wavelength. The percentage hemolysis was calculated based on the following equation, where A is the absorbance of the corresponding sample:

(2) Hemolysis %=Asample−AbufferATriton X100−Abuffer

### 2.6. In Vitro Benzydamine Release

The drug release behavior of benzydamine hydrochloride from κ-CA and κ-CA-SH was investigated by a diffusion membrane model [18]. In brief, the formulations were separated from the release medium by a semipermeable membrane. First, 29 mg of κ-CA or κ-CA-SH and 1 mg of benzydamine were dissolved in 1mL 100 mM phosphate buffer pH 6.8 and transferred into dialysis tubes (SpectraPor Float-A-Lyzer G2 Dialysis Device MWCO 0.5–1 kD) and dialyzed against 9 mL of 100 mM phosphate buffer pH 6.8 in 50 mL falcon tubes while shaking at 300 rpm and 37 °C. A 200 μL sample was taken from the release medium every hour and replaced by a fresh buffer (37 °C). The in vitro drug release was analyzed spectrophotometrically at 306 nm (Tecan Spark multimode microplate reader, Grödig, Austria) [19] as absorbance scans showed the maximum at this wavelength. The concentrations were calculated using a benzylamine hydrochloride standard curve at various concentrations [20].

### 2.7. Tensile Studies on Porcine Mucosa

Tensile studies were conducted using a texture analyzer (TA.XTplus Texture analyzer, Stable Micro Systems, Godalming, UK), examining the adhesive properties of freshly excised porcine buccal mucosa [17,21]. With this measurement, the maximum detachment force (MDF, the force needed for separating test disks from the mucosa) and the total work of adhesion (TWA, the area under the force versus distance curve) were recorded and calculated. Therefore, freshly excised porcine buccal mucosa, with a thickness of about 5 mm, was cut into pieces of approximately 16 cm^2^ and fixed between two acrylic glass plates belonging to the corresponding test rig (A/MUC, Stable Micro Systems, Godalming, UK). The polymer test disks were attached with a double-sided adhesive tape fixed on a cylindrical probe adapter with a diameter of 10 mm. During the experiment, the probe adapter was lowered with a speed of 0.5 mm/s to the mucosa, and a trigger force of 0.2 N, an applied force of 0.1 N, and a contact time of 10 s and 5 min were used before a detachment speed of 2.0 mm/s was applied. During the experiment, the mucosa was kept hydrated with 100 µL of artificial saliva pH 6.8 comprising 1.2 g/L of potassium chloride, 0.85 g/L of sodium chloride, 0.13 g/L of calcium chloride, 0.13 g/L of potassium phosphate dibasic as well as 0.05 g/L of magnesium chloride. Data analysis was carried out using Exponent software.

Tensile studies were also performed using pieces of the same size of freshly excised porcine small intestinal mucosa, as already described above. For this experiment, a force of 0.5 N was applied for a contact time of 5 min. Subsequently, the probe adapter was moved up with a speed of 0.1 mm/s. The MDF and TWA were detected, and data analysis was implemented by Exponent software [22].

### 2.8. Rheological Investigations

Experiments were implemented using a cone-plate combination rheometer (Haake Mars Rheometer, 40/60, Thermo Electron GmbH, Karlsruhe, Germany; Rotor: C35/1°, *D* = 35 mm) while sustaining a constant temperature of 37 °C and setting the gap between cone and plate to 0.052 mm. Within the region of linear viscoelasticity, oscillatory stress sweep measurements were carried out with shear stress in the range of 0.01–50.0 Pa with a constant frequency of 1 Hz. Parameters such as dynamic viscosity (*η*), elastic modulus (*G*′), as well as viscous modulus (*G*″) were obtained [23,24].

Briefly, freshly excised porcine small intestinal mucosa received from a local abattoir was cut longitudinally to collect porcine mucus by scrapping it off from the underlying tissue. After that, the mucus was purified via the addition of 5 mL of 0.1 M sodium chloride solution to 1 g of mucus and stirred for 1 h at 4 °C homogenizing the suspension, followed by centrifugation at 10,400 g for 2 h at 10 °C. This purification step was repeated in the following, and the supernatant was discarded, obtaining the final purified mucus. Hence, samples were stored at −20 °C before use.

For rheological measurements, either κ-CA or κ-CA-SH were dissolved in 100 µL of 100 mM phosphate buffer pH 6.8 in a concentration of 0.25%, 0.50%, and 1.00% (*m*/*v*). Polymer solutions and porcine mucus were mixed and homogenized in a ratio of 1:5 (*v*/*m*). Measurements were carried out after incubation of the mixtures for 3 h at 37 °C, evaluating the viscoelastic characteristics.

### 2.9. In Vitro Mucoadhesion Studies on Porcine Mucosa

Unmodified κ-CA or κ-CA-SH were labeled with FITC as previously described [25]. Briefly, 500 mg of the polymer was dissolved in 100 mL of DMSO, and 6.25 mg of FITC, as well as 375 µL of DBTDL, were added. The mixture was stirred in the dark for 24 h at room temperature. The labeled polymers were purified by dialysis against demineralized water at pH 5.5 in the dark using ZelluTrans MWCO 3500 Da dialysis tube until no fluorescence was detectable in the dialysis medium.

Mucoadhesive properties of polymers were evaluated using freshly excised porcine buccal mucosa being cut longitudinally, obtaining pieces in a size of approximately 4 × 2 cm. The mucosal tissues were fixed on previously halved falcon tubes (50 mL) and placed in a thermostatic chamber (Heratherm Oven, Thermofisher Scientific, Dreieich, Germany) at an angle of 45 °C, and the measurements were carried out at 37 °C and 100% relative humidity. The mucosa was rinsed for 15 min with artificial saliva pH 6.8 comprising 1.2 g/L of potassium chloride, 0.85 g/L of sodium chloride, 0.13 g/L of calcium chloride, 0.13 g/L of potassium phosphate dibasic as well as 0.05 g/L of magnesium chloride, with a flow rate of 1 mL/min utilizing a peristaltic pump (Ismatec, IPC, High Precision Multichannel Dispenser, Richmond Scientific, Lancashire, UK). After that, 10 mg of FITC-labeled polymers were applied to the mucosa and incubated for 5 min. Then, the mucosa was continuously rinsed with artificial saliva pH 6.8 with a flow rate of 1 mL/min, and samples were collected every 30 min up to 180 min. The collected samples were centrifuged at 13,400 rpm for 10 min (MiniSpin^®^, Eppendorf AG, Hamburg, Germany) and 0.1 mL of each sample was transferred to the Spark^®^ multifunctional microplate reader (Tecan Spark multimode microplate reader, Grödig, Austria, GmbH), and fluorescence intensity was measured at an emission wavelength of 530 nm and an excitation wavelength of 490 nm. All experiments were performed in triplicate. For control purposes, 30 mL of artificial saliva pH 6.8 flowing down the mucosa without the CAs were collected, and 10 mg of FITC-labeled κ-CA or κ-CA-SH was dissolved therein and served as a 100% reference value for calculation.

Mucoadhesion was also studied on porcine small intestinal mucosa. The measurement was carried out similarly as described previously using freshly excised porcine small intestinal mucosa. The mucosa was cut longitudinally, obtaining approximately 4 × 2 cm pieces. The mucosal tissues for this measurement were fixed on the halved falcon tubes, then placed at an angle of 45 °C, and the measurements were carried out at 37 °C and 100% relative humidity. The tissues were rinsed with 100 mM phosphate buffer pH 6.8 with a flow rate of 1 mL/min for 15 min. Afterward, 10 mg of FITC-labeled polymers were applied to the mucosa and incubated for 5 min. Then, the mucosa was continuously rinsed with 100 mM phosphate buffer pH 6.8 with a flow rate of 1 mL/min, and samples were collected every 30 min up to 180 min. The collected samples were centrifuged at 13,400 rpm for 10 min (MiniSpin^®^, Eppendorf AG, Hamburg, Germany) and 0.1 mL of each sample was transferred to the Spark^®^ multifunctional microplate reader (Tecan Austria, GmbH), and fluorescence intensity was measured at an emission wavelength of 530 nm and an excitation wavelength of 490 nm. All experiments were performed in triplicate. For control purposes, 30 mL of 100 mM phosphate buffer pH 6.8 flowing down the mucosa without the CAs were collected, and 10 mg of FITC-labeled κ-CA or κ-CA-SH was dissolved therein and served as a 100% reference value for calculation.

### 2.10. Statistical Data Analysis

Statistical data analysis was carried out employing Student’s *t*-test while setting *p* < 0.05 as the minimal level of significance analyzing two groups in contrast to one-way ANOVA for more than two groups. Moreover, the Bonferroni test being a type of multiple comparison test as post hoc analysis was implemented. Results were expressed as means of at least triplicates ± SD.

## 3. Results and Discussion

### 3.1. Synthesis and Characterization of Highly Thiolated κ-CA

**Scheme 1:**
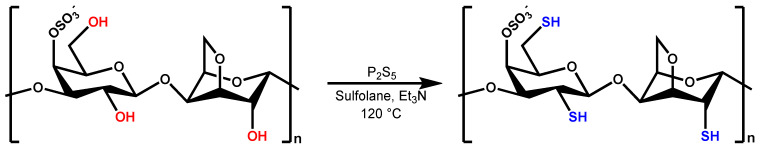
Schematic representation of the synthesis route of highly thiolated kappa-carrageenan (κ-CA-SH) via thiolation with phosphorus pentasulfide.

The parental polymer, κ-CA was thiolated in order to enhance its mucoadhesive properties and prolong its residence time on mucosal membranes. Among other thiolation possibilities, the direct conversion of hydroxyl to thiol groups is most advantageous, as the overall structure of the polymer is altered to the lowest extent. In this work, κ-CA was reacted with phosphorous pentasulfide resulting in the thiolated polymer as illustrated in Scheme 1.

**Figure 1:**
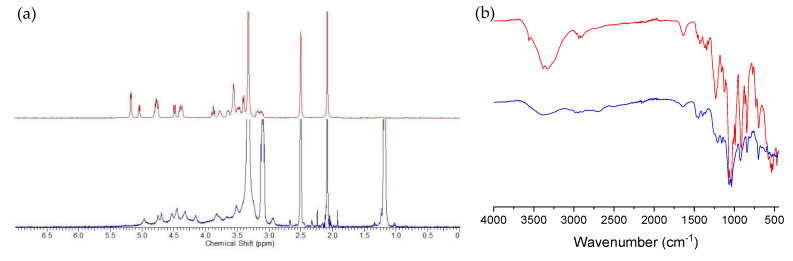
400 MHz ^1^H NMR (**a**) and FTIR (**b**) spectra of the native (red) and thiolated (blue) κ-carrageenan of this work.

The highly thiolated κ-CA structure was confirmed by ^1^H NMR analyses and FTIR (Figure 1). The original signals of native κ-CA broadened after thiolation, and a new peak at 1.20–1.15 ppm appeared, belonging to the sulfhydryl groups. The product’s Fourier-transform infrared (FTIR) spectra also confirmed thiolation of κ-CA, as the strong -O-H stretching peak at 3200–2700 cm^−1^ was decreased, while a new broad peak for the -S-H stretching vibrations between 2650 and 2800 cm^−1^ appeared. The amount of free and oxidized thiol groups of κ-CA-SH was quantified via Ellman’s and disulfide bond tests, respectively. The product contained free thiol groups in 1213.88 ± 52 µmol/g concentration, while 798.33 ± 113 µmol/g disulfide bonds were determined.

### 3.2. Stability of Thiol Groups

**Figure 2:**
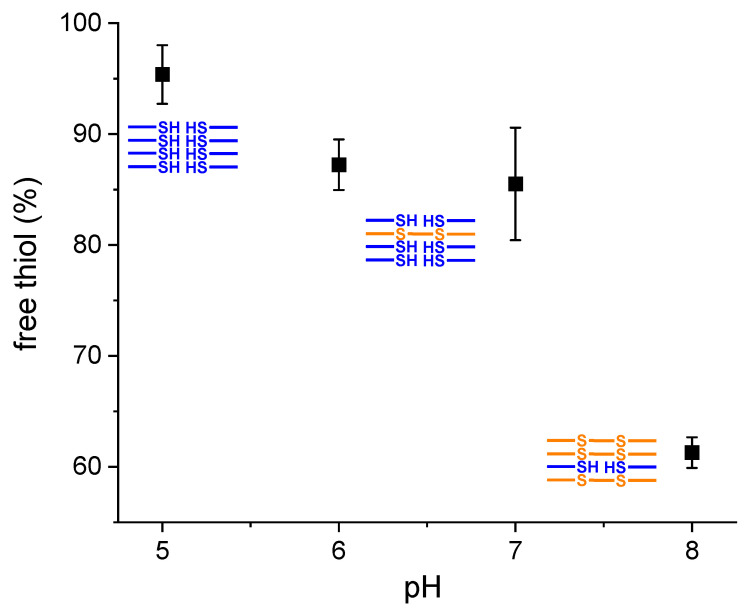
Stability of the thiol groups of κ-CA-SH, as a measure of remaining free thiol groups at indicated pH values. Measurements were carried out at 37 °C, in concentration of 1.25 mg/mL for 4 h. Indicated values are illustrated as means ± SD (*n* ≥ 3).

Stability of thiol groups was measured at different pH values. The amount of free thiols was measured after stirring κ-CA-SH in various buffer systems for 4 h. As shown in Figure 2, at pH 5, almost all thiols remained intact, and no significant disulfide formation occurred. At pH 6 and 7, 87.5 ± 2.3% and 85.2 ± 5.1% of the thiols remained in their free sulfhydryl form, respectively. In contrast, at pH 8, only 61.2 ± 2.3% of thiols remained stable. This lower stability at higher pH can be explained by the higher concentration of thiolate anions being responsible for disulfide formation.

### 3.3. Evaluation of Cytotoxicity

**Figure 3:**
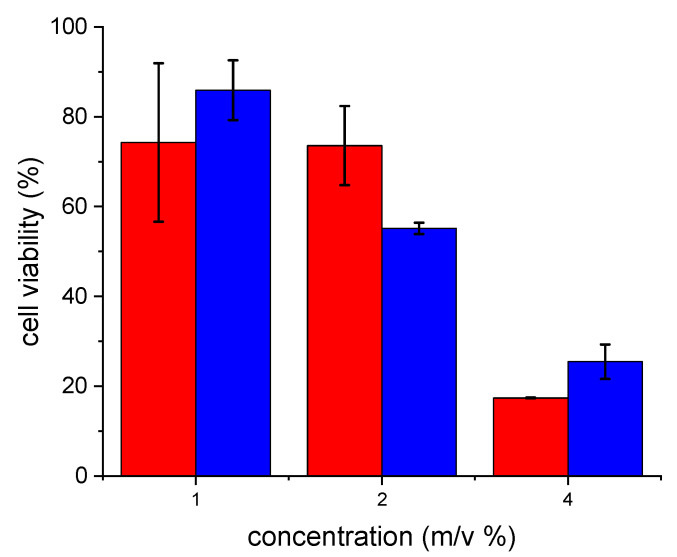
Cell viability of Caco-2 cells after 24 h of incubation at 37 °C in a concentration of 1%, 2%, and 4% (*m*/*v*). Native κ-CA = red bars, highly thiolated κ-CA-SH = blue bars. Indicated values are illustrated as means ± SD (*n* ≥ 3).

A resazurin assay was performed to evaluate cellular metabolic activity as viable cells can metabolize resazurin to its reduced form, resorufin. As displayed in Figure 3, no cytotoxic potential of native and thiolated κ-CA could be detected within 24 h of incubation in a concentration of 1% (*m*/*v*). Up to this concentration, cell viability of κ-CA-SH was close to or even higher than that of native κ-CA, incubated on a Caco-2 cell monolayer within 24 h. In higher concentrations (2% and 4% (*m*/*v*)), a general decrease in cell viability could be observed within 24 h of incubation for both κ-CA, as expected.

### 3.4. Hemolysis Test

**Figure 4:**
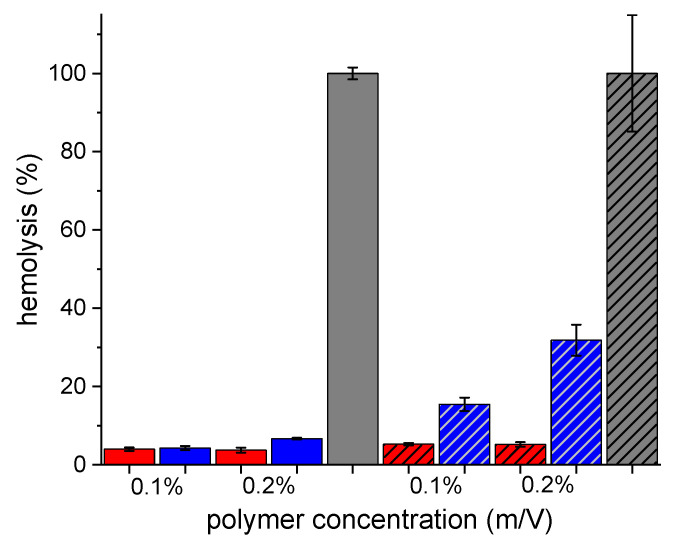
Hemolysis of red blood cells caused by 0.1% (*m*/*v*) and 0.2% (*m*/*v*) native (red columns) and thiolated (blue columns) κ-CA, as well as Triton X-100 after 1 h (columns without pattern) and 3 h (columns with striped pattern) of incubation at 37 °C. Indicated values are illustrated as means ± SD (*n* ≥ 3).

Hemolysis test is an in vitro assay that gives information about the membrane toxicity of the compounds κ-CA and κ-CA-SH. As depicted in Figure 4, no significant hemolysis of either κ-CA or κ-CA-SH was found after 1 h of incubation in concentrations of 0.1 and 0.2% (*m*/*v*). After 3 h of incubation with red blood cells, a slightly increased hemolysis was found for κ-CA-SH, especially at the higher concentration. This increased hemolytic activity, compared to native κ-CA, can be explained the presence of thiol groups on this polymer, which can form disulfides with exofacial thiols of membrane proteins, resulting in some minor membrane damage. Based on cytotoxicity and hemolysis studies, κ-CA-SH is considered as safe at low concentrations.

### 3.5. In Vitro Benzydamine Release

Benzydamine hydrochloride is a locally acting nonsteroidal anti-inflammatory drug (NSAID) with anti-inflammatory, analgesic, antipyretic, as well as anesthetic activity for pain relief and anti-inflammatory treatment of mouth and throat [26,27]. The poor retention at the site of application of marketed solutions used as mouthwash and spray or lozenge formulations of benzydamine is, however, still a major drawback making frequent applications, in the necessary range of three to six times per day [26,28,29]. Therefore, developing novel formulations with sustained release and mucoadhesive properties would have high practical relevance while reducing the application frequency [30].

**Figure 5:**
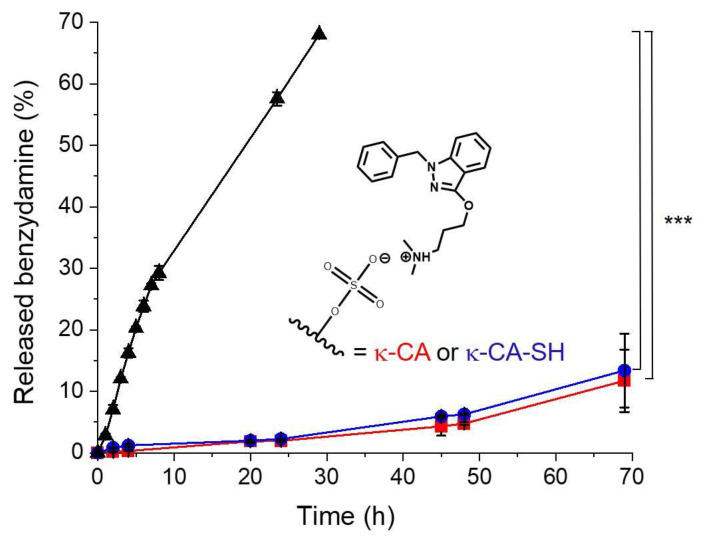
In vitro benzydamine release from solution based on native κ-CA (red dots) and κ-CA-SH (blue dots) in comparison with free benzydamine solution (black triangles) with the schematic structure of the polymer-benzydamine ionic complex. Studies were conducted in artificial saliva (pH 6.8) at 37 °C. Indicated values are depicted as means ± SD (*n* ≥ 3) (*** *p* < 0.001).

Benzydamine hydrochloride is a positively charged drug that can be ionically linked to negatively charged CA. Utilizing an aqueous solution of κ-CA and κ-CA-SH together with benzydamine, the release of the drug was investigated via dialysis against artificial saliva (pH 6.8), and the results are shown in Figure 5. No significant difference between κ-CA and κ-CA-SH was found for this system; in both cases slow release of benzydamine was detected. After 24 h, only 1.95 and 2.25% of the added drug was released from κ-CA and κ-CA-SH, which increased further to 4.75 and 6.27% after 48 h, respectively. The similar drug release profile of κ-CA and κ-CA-SH can be explained by the anionic character of these polymers, responsible for ionic interactions with the positively charged benzydamine hydrochloride, that were not altered due to thiolation. In comparison, a faster release of benzydamine from an aqueous solution was determined, reaching almost 27% within 6 h and 60% after 24 h. In order to investigate the role of ionic interactions in sustained drug release, the release of sodium fluorescein, an anionic model compound, was also tested with and without κ-CA and κ-CA-SH. Due to the lack of ionic interactions between the polymers and the model drug, no differences in the release profiles with or without polymer was detected; in all cases between 38.2 ± 4.4% and 38.5 ± 1.0% of the anionic compound was released within 7 h.

Since the dialysis membrane causes per se a sustained release of the drug to the acceptor compartment, these results cannot be extrapolated to the in vivo situation. Nonetheless, they provide strong evidence for ionic interactions between the polymer and the drug, as a more sustained drug release was observed in the presence of polymer. This slow release is advantageous for such mucoadhesive formulations, resulting in a long-lasting local effect and guaranteeing a constant release of benzydamine to the affected areas in the oral cavity [31].

In comparison to previous approaches, using mucoadhesive polymers, such as modified celluloses, Carbopol, or polycarbophil, for the design of buccal delivery systems for benzydamine hydrochloride, a more sustained drug release was achieved [32,33,34,35]. This sustained release of benzydamine hydrochloride in case of κ-CA-SH makes the polymeric carrier with potential mucoadhesive properties attractive for buccal applications.

### 3.6. Tensile Studies on Porcine Mucosa

**Figure 6:**
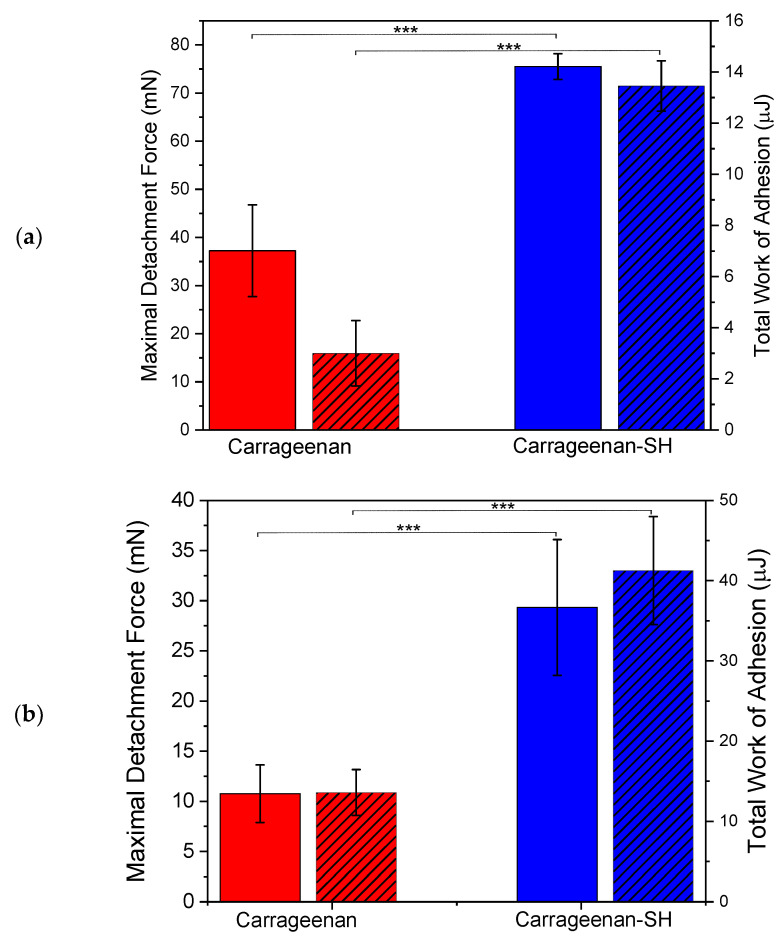
Tensile studies on (**a**) porcine buccal mucosa and (**b**) porcine small intestinal mucosa. Investing tensile strength via measuring MDF (columns without pattern) apart from TWA (columns with striped pattern) of test disks using a texture analyzer. Native κ-CA = red bars, highly thiolated κ-CA-SH = blue bars. Indicated values are presented as means ± SD (*n* ≥ 3) (*** *p* < 0.001).

The thiolation of κ-CA allows this modified polymer to form disulfide bonds with the mucosal layer. Assessing the mucoadhesive features of highly thiolated κ-CA-SH for buccal application, tensile studies were performed using κ-CA and κ-CA-SH test discs on freshly excised porcine buccal mucosa to investigate the adhesive properties of the two polymers. Since Baus et al. could show a high correlation of results obtained with various polymers via tensile studies with their mucoadhesive properties in the oral cavity of human volunteers, this experimental setup is likely highly predictive [22]. In the presence of κ-CA-SH, maximal detachment force (MDF) and total work of adhesion (TWA) increased significantly, as displayed in Figure 6a. After 10 s of contact time, κ-CA-SH could achieve a 2.0-fold improved MDF and 4.5-fold higher TWA in contrast to κ-CA. The same trend could be observed after 5 min contact time, whereby κ-CA-SH displayed a 2.2-fold and 7.7-fold higher MDF and TWA, respectively.

Tensile studies were also performed on intestinal mucosa (Figure 6b). Generally, slightly different MDF and TWA values were reached than in case of porcine buccal mucosa due to the different microscopic anatomy of the two tissues [32,33], as anticipated from previous studies [34]. In this case, a 2.7-fold higher MDF and a 3.0-fold improved TWA compared to the corresponding unmodified κ-CA confirmed the strong adhesion between the intestinal mucosa and the thiolated polymer.

In cases of both buccal and intestinal mucosa, higher MDF and TWA values were determined for κ-CA-SH than in previous approaches using other mucoadhesive polymers, for example, poly(acrylic acid) or modified celluloses, for benzydamine delivery [35,36,37]. The enhanced mucoadhesive properties, in the case of κ-CA-SH, are due to the thiolation of the polymer, which can form strong covalent bonds with the mucus glycoproteins. In case of previously described acidic polymers, mucoadhesion occurs through weaker secondary interactions, such as hydrogen bonding with the mucosal membrane. The investigated drug, benzydamine hydrochloride, is also approved for vaginal administration in some countries [26]. Nonetheless, due to the similarity of buccal and vaginal mucosa [38,39], we did not perform tensile studies on vaginal mucosa.

### 3.7. Rheological Investigations

**Figure 7:**
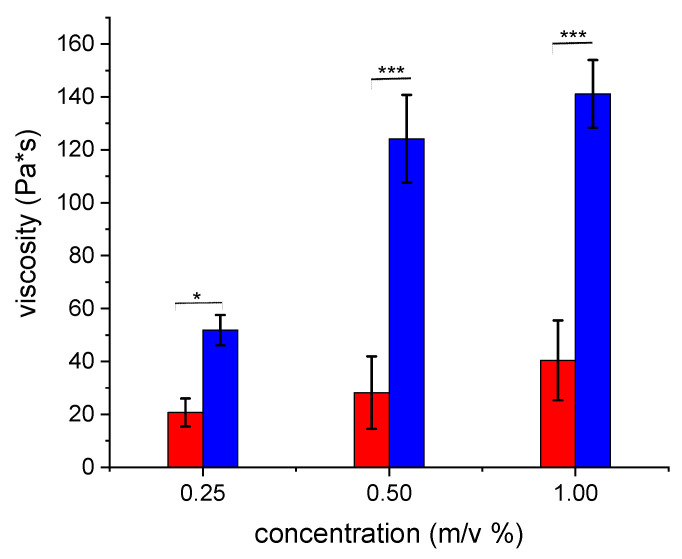
Rheological behavior of native κ-CA (red bars) and highly thiolated κ-CA-SH (blue bars) in porcine mucus in a ratio of 1:5 in a concentration of 0.25%, 0.5%, and 1% (*m*/*v*) in 100 mM phosphate buffer pH 6.8 determining dynamic viscosity (*ƞ*) within 3 h of incubation at 37 °C. Indicated values are outlined as means ± SD (*n* ≥ 3) (*** *p* < 0.001, * *p* < 0.05).

The thiolated polymer is expected to introduce new cross-linking points in the mucus through thiol-disulfide exchange reactions with the cysteine moieties of mucus glycoproteins [40]. Hence, the viscosity of this mixture should be higher than that of the original mucus or its mixture with the non-thiolated polymer. For κ-CA-SH, an increase in dynamic viscosity (*η*) with mucus was shown in all employed concentrations, as depicted in Figure 7. Generally, the higher the concentration of κ-CA-SH, the more new cross-linking points were formed, resulting in higher viscosity. At a carrageenan concentration of 0.25% (*m*/*v*), a 2.5-fold increase in viscosity was determined, whereas at polymer concentrations of 0.50% and 1.00%, an up to 4.4-fold increase in viscosity, compared to native κ-CA/mucus mixtures was observed.

### 3.8. In Vitro Mucoadhesion Studies on Porcine Mucosa

The prolonged mucosal residence time of thiolated excipients is auspicious for drugs used for local treatment. Therefore, retention of native κ-CA and κ-CA-SH on porcine buccal mucosa was compared in order to test and evaluate prolonged mucosal residence time after thiolation.

**Figure 8:**
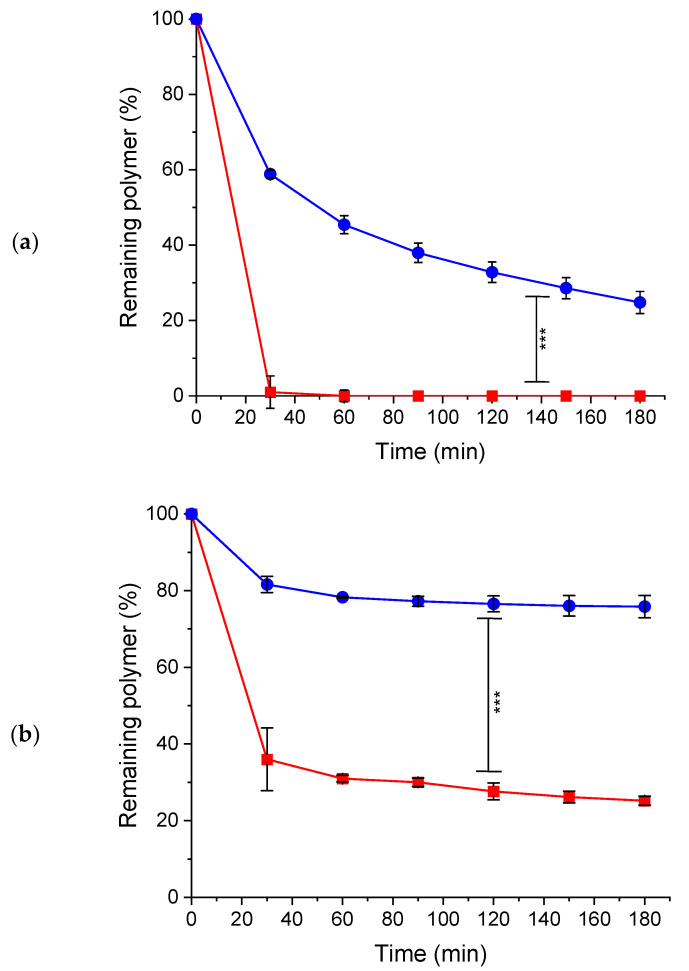
Percentage of remaining κ-CA (red squares) and κ-CA-SH (blue circles) on (**a**) porcine buccal mucosa and (**b**) porcine small intestinal mucosa, continuously rinsed with 100 mM phosphate buffer pH 6.8 and artificial saliva, respectively. The data are shown as mean ± SD (*n* = 3) (*** *p* < 0.001).

Fluorescein isothiocyanate modified κ-CA and κ-CA-SH were used for the retention studies. The removal of the labeled polymers from the mucosal surface was measured by rinsing with artificial saliva pH 6.8. As shown in Figure 8a, native κ-CA was removed from buccal mucosa within 30 min. The κ-CA-SH showed higher mucoadhesive properties on the buccal mucosa. After 30 min, around 40% of the added polymer was rinsed off, while after 180 min 75% of the labeled κ-CA-SH was removed.

Based on the tensile studies, a slightly different residence time on the intestinal than on buccal mucosa was expected for both κ-CA and κ-CA-SH. Indeed, 60% of the native carrageenan was rinsed off within the first 30 min, and it was further removed, as only 25% of κ-CA remained on the mucosal layer after 180 min (Figure 8b). This indicates higher intestinal mucoadhesion than in case of buccal mucosa, where all the applied κ-CA was removed within 30 min. A slow and minor removal was detected for κ-CA-SH, and almost 76% of this product remained on the mucosal surface after 3 h of rinsing with PBS buffer at pH 6.8.

The prolonged residence time of κ-CA-SH on both mucosal membranes can be explained by the high degree of thiolation; consequently, numerous stable disulfide bonds are formed between the cysteine-rich mucus glycoproteins and the thiolated polymer, anchoring the excipient to the mucus layer. Compared to the also mucoadhesive native κ-CA, the enhanced mucoadhesive properties of κ-CA-SH, together with the sustained release of benzydamine hydrochloride drug, make this thiolated polymer attractive for buccal applications.

## 4. Conclusions

Highly thiolated κ-carrageenan κ-CA-SH) was synthesized by reacting the native polymer with phosphorous pentasulfide. The success of the reaction was verified by ^1^H NMR and FTIR spectroscopy, as well as Ellman’s test, and the stability of the product was confirmed at and below pH7. Benzydamine hydrochloride, ionically linked to κ-CA-SH, showed slow and sustained release profiles from solution. The cytotoxicity and hemolysis of the modified κ-CA remained relatively low at low concentrations, while its mucoadhesive properties increased highly compared to native κ-CA. Up to 2.7-fold higher maximum detachment force and 7.7-fold higher total work of adhesion confirmed the enhanced mucoadhesion after thiolation. The viscosity of mucus increased up to 4.4-fold, while also highly prolonged mucosal residence time was detected for the κ-CA-SH on both buccal and intestinal mucosa, compared to the unmodified polymer. According to these results, with a high degree of modification, κ-CA-SH could be a potential mucoadhesive excipient for intraoral drug delivery by prolonging the residence time of cationic drugs on mucosal membranes and thus reducing the dosage frequency.

## Data Availability

Data available on request to the corresponding author.
